# Iatrogenic Blood Loss in Very Low Birth Weight Infants and Transfusion of Packed Red Blood Cells in a Tertiary Care Neonatal Intensive Care Unit

**DOI:** 10.3390/children8100847

**Published:** 2021-09-25

**Authors:** Ahmed Aboalqez, Philipp Deindl, Chinedu Ulrich Ebenebe, Dominique Singer, Martin Ernst Blohm

**Affiliations:** Division of Neonatology and Pediatric Intensive Care Medicine, University Children’s Hospital, University Medical Center Hamburg-Eppendorf, Martinistr. 52, 20246 Hamburg, Germany; a.aboalqez@uke.de (A.A.); p.deindl@uke.de (P.D.); c.ebenebe@uke.de (C.U.E.); d.singer@uke.de (D.S.)

**Keywords:** VLBW neonate, blood sampling, blood transfusion, iatrogenic blood loss

## Abstract

An adequate blood volume is important for neonatal adaptation. The study objective was to quantify the cumulative iatrogenic blood loss in very low birth weight (VLBW) infants by blood sampling and the necessity of packed red cell transfusions from birth to discharge from the hospital. In total, 132 consecutive VLBW infants were treated in 2019 and 2020 with a median birth weight of 1180 g (range 370–1495 g) and a median length of stay of 54 days (range 0–154 days) were included. During the initial four weeks of life, the median absolute amount of blood sampling was 16.5 mL (IQR 12.3–21.1 mL), sampling volume was different with 14.0 mL (IQR 12.1–16.2 mL) for non-transfused infants and 21.6 mL (IQR 17.5–29.4 mL) for transfused infants. During the entire length of stay, 31.8% of the patients had at least one transfusion. In a generalized logistic regression model, the cumulative amount of blood sampling (*p* < 0.01) and lower hematocrit at birth (*p* = 0.02) were independent predictors for the necessity of blood transfusion. Therefore, optimized patient blood management in VLBW neonates should include sparse blood sampling to avoid iatrogenic blood loss.

## 1. Introduction

An adequate circulatory volume is essential in perinatal transition and during the neonatal period [1]. During a regular physiological vaginal delivery, a placental blood transfusion to the newborn occurs in the period after the birth of the child, before the umbilical cord is clamped. 

At delivery of a mature neonate, 70 mL/kg blood, referring to the infant’s body weight, are in the infant, and 35 mL/kg in the placenta [2]. With delayed cord clamping, placental blood is shifted to the newly born infant resulting in a blood volume of approximately 93 mL/kg body weight after three minutes [2]. This perinatal auto-transfusion of placental blood into the neonate appears to be a biologically useful mechanism to help perinatal adaptation [1,2,3,4,5], particularly for premature infants with their physiologically low absolute amount of blood volume. 

Neonatal blood volume can be preserved by sparse blood sampling for diagnostic purposes. Iatrogenic neonatal blood loss in association with blood sampling [6,7,8,9,10,11,12,13] has been an issue for decades. Several studies have actually quantified the iatrogenic blood loss in VLBW neonates [6,7,8,9,10,11]. The amount of blood taken from ELBW and VLBW infants during the first 28 days of life has been reported as 31 mL/kg body weight in 1981 [6], 50.3 mL/kg body weight [7] in 1988, and 24.2 mL/kg body weight in 2019 [8]. In a recent study published in 2020, the average cumulative 28 d blood loss in ELBW neonates with an umbilical artery catheter (UAC) in place was 69 mL (108 mL/kg) while the average cumulative blood loss without UAC in place was 32 mL (43 mL/kg) [9]. Different studies quantifying iatrogenic neonatal blood loss have in common, that smaller neonates (ELBW and VLBW) have a relatively higher amount of blood sampling in mL/kg body weight than larger neonates [6,7,8,9,10,11]. Still, iatrogenic blood loss by blood sampling is one of the main factors for anemia in VLBW infants, leading to the necessity of packed red cell transfusions. Even in recent publications, approximately 50% of VLBW neonates with a normal hematocrit at birth require at least one transfusion during their hospital stay [14]. This study aimed to quantify cumulative iatrogenic blood losses in VLBW neonates and correlate them to demographic and clinical outcome parameters and to blood transfusions. With this study, we want to raise awareness for good neonatal patient blood management [15] by preserving blood volume, starting at initial stabilization or resuscitation and then throughout the entire length of hospital stay of this patient population. 

## 2. Materials and Methods

This observational study was conducted as a retrospective single-center study at a tertiary referral center (University Hamburg-Eppendorf Medical Center, Hamburg, Germany). We included VLBW and ELBW infants born in the University Medical Center Hamburg-Eppendorf during 2019 and 2020 into the analysis. Transfusion triggers for packed red cells were restrictive [16,17,18], based on the national guidelines, generally following the restrictive arm of the ETTNO trial [18]. Neonatal outcome parameters were defined as follows: Bronchopulmonary dysplasia (BPD) as additional oxygen demand at a corrected age of 36 weeks, intraventricular hemorrhage (IVH) in cases with IVH grade 3 or 4, necrotizing enterocolitis (NEC) as Bell stage 2 and 3, retinopathy of prematurity (ROP) was defined by the necessity of intraocular vascular endothelial growth inhibitor (VEGF-inhibitor) administration or laser treatment. Blood sampling, transfusion, and clinical and outcome data were extracted from the electronic patient data management system (PDMS ICM, Dräger, Lübeck, Germany). For individual types of laboratory tests (e.g., full blood count, clinical chemistry, drug level monitoring, clotting studies, blood gas analysis, blood samples for crossmatching packed red cells, neonatal metabolic screening, genetic testing) the amount of blood typically required in the setting of the neonatal intensive care unit (NICU) and blood losses in association with vascular access were defined as follows: blood gas analysis 100 µL, newborn screening 250 µL, full blood count 750 µL, infection screen including IL6 and CRP 750 µL, extended blood tests including liver and renal function tests 1000 µL, blood culture 500 µL, drug levels 750 µL, clotting studies 1300 µL, blood losses with the establishment of venous or arterial access 750 µL. The cumulative volume of all blood samples and losses for each patient during the entire hospital stay was then calculated based on the laboratory results in the PDMS. In total, 164 patients were eligible. Patients transferred to other hospitals or in-house wards not equipped with the electronic PDMS before discharge at home were excluded. Subsequently, 132 VLBW neonates were included in the analysis. 

For statistical analysis with appropriate tests (Chi-Square test statistics and Fisher‘s exact test, pairwise Pearson correlation, t-test, generalized logistic regression model) R (Version 4.0.3, R Core Team, 2020) and SPSS (version 20, IBM Inc., Chicago, IL, USA) were used. Data collection and anonymized data handling were in concordance with the local Review Board (Ethik-Kommission Ärztekammer Hamburg, Germany, WF-075/21, 29 March 2021). 

## 3. Results

### 3.1. Patients

Data from 132 VLBW infants were included. Table 1 shows the demographic and outcome parameters of the studied sample cohort.

### 3.2. Blood Transfusion

The rate of patients receiving at least one packed red cell transfusion during their hospital stay was 31.8%. The cumulative blood transfusion volume during the entire hospital stay was 33.5 mL on average (IQR 20–53.75 mL) in patients who received at least one transfusion. Patients with and without a blood transfusion were demographically different (Table 2) with a higher birth weight and gestational age and a shorter duration of stay in non-transfused patients. 

### 3.3. Blood Sampling 

The absolute amount of blood sampling is shown in Table 3. The median absolute amount of blood sampling [mL] during the initial four weeks of life was 16.5 mL (IQR 12.3–21.1 mL). Median sampling volume and median gestational age were different with 14.00 mL (IQR 12.05–16.20 mL)/gestational age 30 + 2 weeks (IQR 28 + 5 − 31 + 5 weeks) for non-transfused infants and 21.60 mL (IQR 17.51–29.40 mL)/26 + 2 weeks (IQR 25 + 3 − 29 + 5 weeks) for transfused infants. 

**Figure 1:**
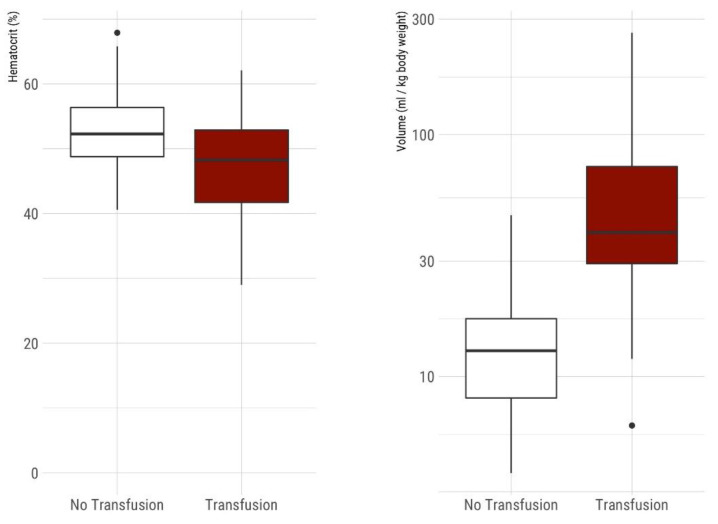
(**Left**) Comparison of hematocrit on admission between non-transfused and transfused VLBW infants. Boxplots show median, IQR, 95% confidence intervals, and outliers. The mean initial hematocrit in non-transfused neonates with 52.6% was significantly higher compared to 47.0% in neonates requiring at least one transfusion during hospital stay (*p* < 0.001). (**Right**) Comparison of cumulative blood sampling volume in VLBW infants during their hospital stay [mL/kg body weight at birth] between non-transfused and transfused VLBW neonates. Boxplots show median, IQR, 95% confidence intervals and outliers, *y*-axis logarithmic scale. Non-transfused neonates (*n* = 90) had significantly less median blood sampling volume 12.78 mL (IQR 8.15–17.35 mL) compared to neonates with one or more red blood cell transfusions during their hospital stay (*n* = 42) with a median cumulative sampling volume of 39.42 mL (IQR 29.33–73.95 mL).

The amount of blood sampling during the total length of stay in relation to birth weight [mL/kg birth weight, median (IQR)] was 16.42 mL (IQR 9.86–32.65 mL) for the whole group; 12.78 mL (IQR 8.15–17.35 mL) for non-transfused infants and 39.42 mL (IQR 29.33–73.95 mL) for transfused infants (Figure 1). 

The mean initial hematocrit in non-transfused neonates with 52.6% was significantly higher compared to 47.0% in neonates requiring transfusion during hospital stay (*p* < 0.001), whereas the hematocrit at discharge was not statistically different between the two groups (31.3% vs. 30.4%, *p* = 0.34) (Figure 1).

**Figure 2:**
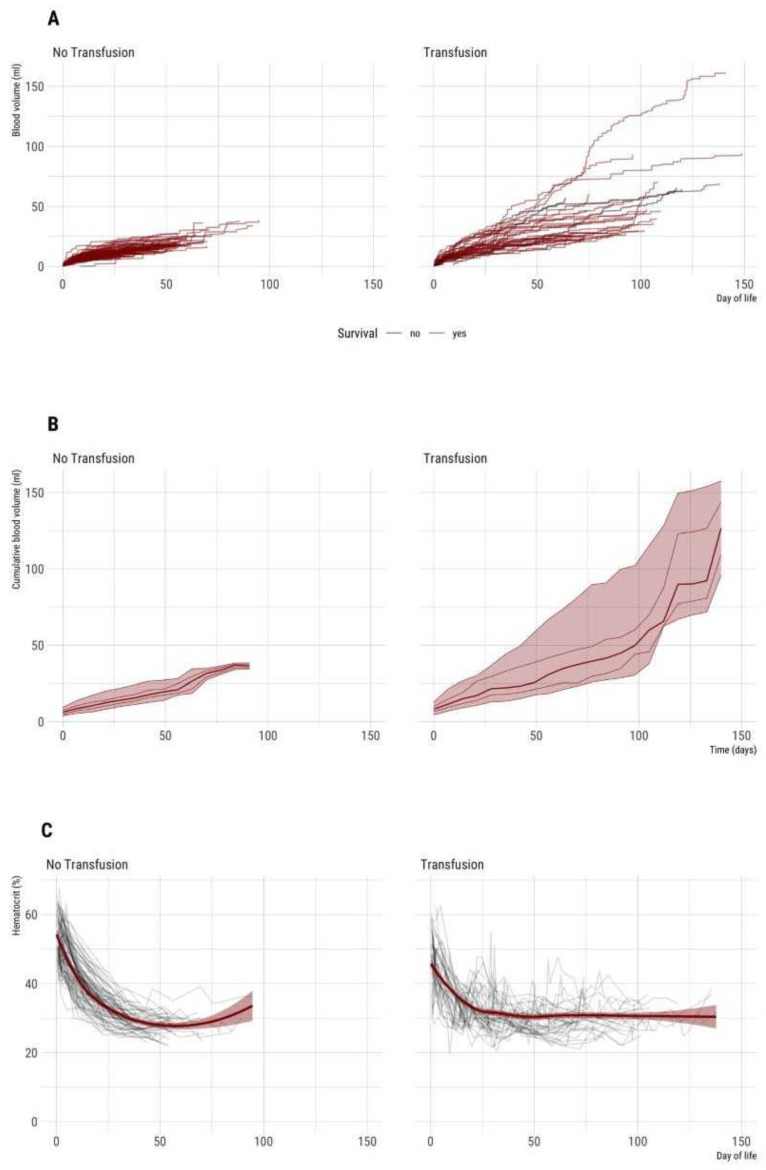
(**A**–**C**) Time course of cumulative blood sampling volume and hematocrit during entire hospital stay (*n* = 132 VLBW neonates included). Left: Non-transfused neonates (*n* = 90). Right: Neonates with one or more red cell transfusions (*n* = 42). (**A**) Absolute cumulative blood sampling volume [mL] over time. Each line represents an individual patient. (**B**) Median and percentiles (percentiles 5, 25, 50 75, 95) are given for a minimum of 3 patients per week, therefore plot truncated at 20 weeks. (**C**) Time course of hematocrit. Each grey line represents an individual patient. The dark red lines show smoothed average hematocrit, the dark red transparent ribbon the 95% CI of mean over time.

The time course of cumulative iatrogenic losses by blood sampling is presented in Table 4 and Figure 2A,B. The cumulative blood sampling volume was significantly different between patients requiring a transfusion and non-transfused patients. The time course of hematocrit is shown in Figure 2C. 

**Figure 3:**
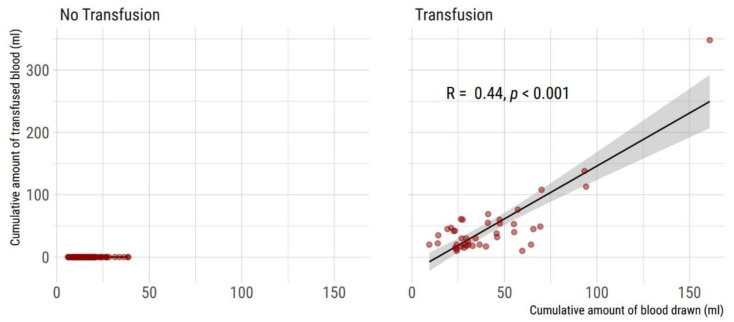
Correlation of cumulative blood sampling volume and cumulative amount of transfused blood during entire hospital stay (*n* = 132 VLBW neonates included). (**Left**) Non-transfused neonates (*n* = 90). (**Right**) Neonates with one or more red cell transfusions (*n* = 42). Two-sided Spearman’s rank correlation coefficient rho and 95% CI is given.

There was a significant correlation between cumulative blood sampling volume and cumulative blood transfusion volume (Figure 3). 

**Figure 4:**
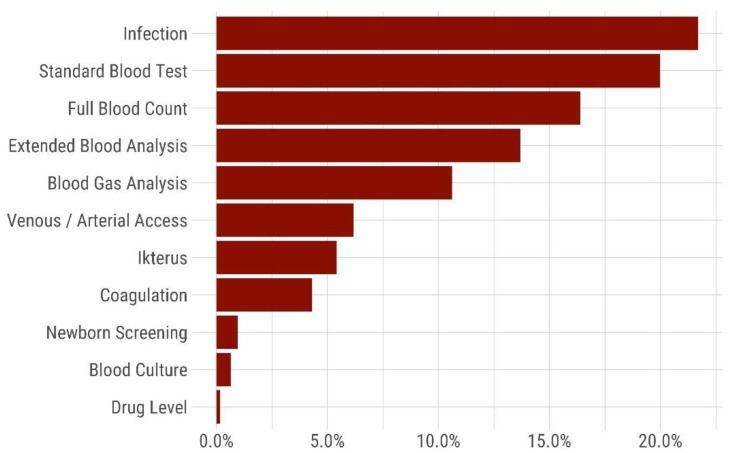
Relative amount [%] of iatrogenic blood loss during the hospital stay (*n* = 132 VLBW included neonates into the analysis). The bars represent the cumulative relative amount of blood loss associated with different laboratory investigations: Infection (sample including IL6), standard blood test (sample with C-reactive protein), full blood count, extended blood analysis (liver and renal function tests), blood gas analysis, losses during vascular access, icterus (bilirubin and liver function test), coagulation (clotting studies), newborn screening (neonatal newborn screening), blood culture, drug level.

An analysis of the relative contribution of iatrogenic blood losses in the study sample of neonates treated in our unit is given in Figure 4. 

### 3.4. Risk Factors for Necessity of Transfusion 

Demographic parameters associated with the necessity for erythrocyte transfusion were birth weight, gestational age, and length of stay, whereas sex, delivery mode, and multiple gestations were not associated with an increased risk of a transfusion requirement. Outcome parameters significantly associated with the necessity for erythrocyte transfusion were sepsis/infection, PDA, NEC, ROP, fatal outcome. In contrast, IVH was not significantly associated with transfusions (Table 4). 

**Figure 5:**
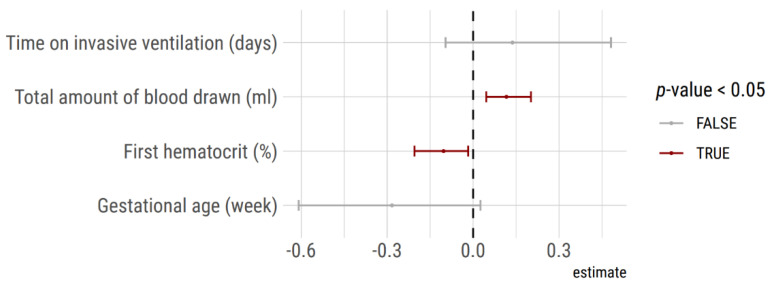
Predictors of transfusion deduced from a generalized logistic model. Cumulative blood sampling volume and hematocrit on admission were significant independent predictors of red blood cell transfusion during the hospital stay in the 132 VLBW included neonates into the analysis.

A generalized logistic model, including time on invasive ventilation, the total amount of blood sampling, first hematocrit, and gestational age was calculated in order to identify independent risk factors for red blood cell transfusion during the hospital stay. The model identified the total amount of blood sampling and first hematocrit as independent predictors for a blood transfusion (Figure 5).

## 4. Discussion

This single-center retrospective study analyzed the amount of iatrogenic blood sampling and subsequent blood loss and requirements for packed red cell transfusions in VLBW infants during their entire hospital stay. The sample cohort comprised a non-selected cross-sectional and consecutive longitudinal group of VLBW neonates. 

The median absolute amount of blood sampling during the initial four weeks of life was 16.5 mL (IQR 12.3–21.1 mL). The cumulative blood sampling volume was comparable to a recent publication on neonatal blood sampling in VLBW infants with a median blood loss of 19.6 mL during the first 28 days [8] and lower than in historical data [6,7]. Our unit strives to keep usage of umbilical vascular catheters restrictive and as short as possible. This may also contribute to a relatively low iatrogenic blood loss [9].

Demographic factors such as lower birth weight, lower gestational age, and longer length of stay were significantly associated with the necessity for erythrocyte transfusion, as well as several adverse neonatal outcome parameters (sepsis/infection, PDA, NEC, ROP, death). Thus, smaller VLBW patients with higher morbidity had a higher risk of receiving a transfusion. 

In addition, a generalized logistic model identified both the cumulative amount of blood sampling (in mL/kg body weight) and the initial hematocrit as significant independent predictors of transfusion in the VLBW infants of our sample cohort. 

The study finding of a higher hematocrit at birth in the group of non-transfused neonates supports the recommendations regarding delayed cord clamping at birth [3,4]. In a meta-analysis, delayed cord clamping increased the hematocrit at birth by 2.73%, resulting in a 10% reduction of red cell transfusions [4]. In our study, the initial hematocrit of non-transfused children was 11.9% higher than in transfused children (52.6% vs. 47.0%). Neonatal data showed an inverse correlation between hemoglobin at birth and the necessity for red blood cell transfusions during the hospital stay in VLBW neonates [14]. 

The study finding of a positive correlation between the amount of cumulative blood sampling and cumulative blood transfusion requirement implies, that all measures to spare blood, which is a valuable and limited resource for the patient, should be implemented [6,7,8,9,10,11,12,13,14,15,19]. Depending on local neonatal and laboratory practice the relative amount of iatrogenic blood losses may be distributed differently compared to our study sample (Figure 4) [10]. Neonatal patient blood management may include the use of umbilical cord or placental blood for admission laboratory values [13]. In addition, storage of maternal blood for ordering and crossmatching of blood products may minimize the amount of blood required for laboratory analyses. Setting point of care devices to minimal blood volumes, use of non-invasive monitoring methods, avoidance of “routine” blood sampling, strict indication, and supervision of blood tests by experienced personnel may help to avoid iatrogenic blood losses [9,12]. Cumulative documentation of blood draw volumes, possibly based on an automated calculation derived from a PDMS system, might also help raise neonatal team awareness in avoiding iatrogenic blood loss.

Perinatal medicine is well aware of the issue of placental transfusion at birth and the possible benefits of delayed cord clamping—providing additional blood volume to neonates [1,2,3,4,5,20,21] as part of initial neonatal management. The intention of this study is to emphasize the importance of the opposite side of the balance, i.e., avoidance of iatrogenic blood loss, especially in VLBW preterm infants as a part of good neonatal blood management [6,7,8,9,10,11,12,13,14,15,19].

Strengths and limitations: This analysis includes the entire hospital stay of a–compared to previously published data [6,7,8,9]—larger sample cohort of VLBW neonates over a longer time course. Blood sampling volumes were retrospectively deduced from the PDMS based on the locally minimally required amount of blood for standard laboratory investigations. This implies a complete cumulative capture of all blood samples, but potentially underestimating blood sampling volumes in case of overfilled blood tubes. The unit policy adheres to restrictive transfusion triggers [16,17,18], but each transfusion was indicated at the physician’s discretion in charge, implying a potential bias in the transfusion trigger. Statistical statements in association with neonatal morbidity are only possible to a limited extent, as the absolute number of patients with complications was low. Nevertheless, we identified the initial hematocrit and the total blood sampling volume as independent predictors for red blood cell transfusion in our patient sample.

## 5. Conclusions

In a patient sample of 132 VLBW neonates, cumulative iatrogenic blood losses during the entire hospital stay and the initial hematocrit were significant independent predictors for the necessity of packed red cell transfusions. Therefore, iatrogenic blood loss should be limited to a minimum in the interest of good patient blood management. 

## Figures and Tables

**Table 1:** Demographic and clinical characteristics of the study population sample.

	Study Population (*n* = 132)
Birth weight [g, median, IQR, range]	1180 (IQR 903–1360, range 370–1495)
Gestational age [weeks, median, IQR, range]	29 + 5 (IQR 27 + 5 to 31 + 2; range 23 + 5 to 36 + 5)
LOS [days, median, IQR, range]	54 (IQR 35–74; range 0–154)
Multiple gestation [*n*; %]	66 (50%)
Female sex [*n*; %]	63 (47.7%)
Delivery mode C-section	121 (91.7%)
Sepsis/Infection [*n*; %]	39 (29.5%)
IVH [*n*; %]	6 (4.5%)
BPD [*n*; %]	6 (4.5%)
ROP [*n*; %]	6 (4.5%)
NEC [*n*; %]	4 (3.0%)
PDA treated medically	28 (21.2%)
PDA treated operatively [*n*; %]	4 (3.0%)
Fatal outcome [*n*; %]	6 (4.5%)

Legend: IQR interquartile range; LOS length of stay; IVH intraventricular hemorrhage (grade 3 and 4); BPD bronchopulmonary dysplasia; ROP retinopathy of prematurity; PDA persistent arterial duct; NEC necrotizing enterocolitis.

**Table 2:** Demographic differences between transfused and non-transfused patients.

Demographic Factor	Transfused Patients (*n* = 42)	Non-Transfused Patients (*n* = 90)	*p* (*t*-Test)
Birth weight [g, median, IQR]	755 (643–943)	1275 (1074–1415)	<0.001
Gestational age [weeks, median, IQR]	26 + 2 (25 + 3 − 29 + 5)	30 + 2 (28 + 5 − 31 + 5)	<0.001
LOS [days, median, IQR]	93 (70–103)	44 (33–59)	<0.001

Legend: IQR interquartile range; LOS length of stay.

**Table 3:** Absolute cumulative blood sampling volume [*n* = 132 VLBW infants].

Postnatal Age [Completed Weeks]	Non-Transfused Patients [mL; Median (IQR)]	Transfused Patients [mL; Median (IQR)]
0	6.18 (4.73–7.35)	8.20 (6.60–9.95)
1	8.65 (7.23–10.53)	11.50 (9.35–14.75)
2	10.33 (8.43–12.69)	14.98 (11.86–18.95)
3	12.45 (10.39–14.01)	17.15 (13.90–26.50)
4	14.00 (12.05–16.20)	21.60 (17.51–29.40)
5	15.50 (13.49–17.71)	22.10 (17.88–32.89)
6	17.68 (15.93–20.64)	23.20 (19.10–35.95)
7	19.45 (17.30–21.25)	25.50 (21.03–35.86)
8	20.90 (18.74–24.84)	30.83 (23.25–41.79)
9	26.45 (21.45–29.45)	34.63 (25.40–44.35)
10	31.45 (29.10–33.25	37.28 (25.11–47.11)
11	33.60 (32.08–35.13)	39.45 (29.50–49.00)
12	36.80 (35.25–37.60)	41.45 (31.38–53.75)
13	36.23 (35.01–37.44)	44.70 (34.70–55.10)
14		49.75 (43.99–60.01)
15		59.90 (45.70–70.05)
16		65.60 (62.65–87.35)
17		89.90 (77.13–123.05)
18		90.10 (78.80–124.13)
19		92.15 (80.75–126.50)
20		126.60 (109.43–143.78)

Legend: IQR interquartile range.

**Table 4:** Chi-Square Test statistics for demographic and outcome factors associated with transfusions, factors ordered in ascending likelihood ratio for the necessity of red blood cell transfusion.

Factor	Likelihood Ratio (Fisher’s Exact Test)	*p*
Sex	0.127	0.852 (n.s.)
Multiple gestation	0.127	0.852 (n.s.)
Delivery mode	0.140	0.740 (n.s.)
IVH	3.216	0.081 (n.s.)
BPD	7.165	0.012 *
Mortality	7.165	0.012 *
ROP	7.165	0.012 *
PDA treated operatively	9.432	0.009 *
NEC	9.432	0.009 *
PDA treated medically	12.892	0.000 *
Sepsis/Infection	30.087	0.000 *
Gestational age	92.743	0.001 *
Length of stay	131.353	0.000 *
Birth weight	136.013	0.000 *

Legend: n.s. no significance; * *p* < 0.05. LOS length of stay; IVH intraventricular hemorrhage (grade 3 and 4); BPD bronchopulmonary dysplasia; ROP retinopathy of prematurity; PDA persistent arterial duct; NEC necrotizing enterocolitis.

## Data Availability

The data are not publicly available due to patient privacy reasons.
